# Toward Better and Healthier Air Quality: Global PM_2.5_ and O_3_ Pollution Status and Risk Assessment Based on the New WHO Air Quality Guidelines for 2021

**DOI:** 10.1002/gch2.202300258

**Published:** 2024-03-26

**Authors:** Jianhua Liu, Chao He, Yajun Si, Bin Li, Qian Wu, Jinmian Ni, Yue Zhao, Qixin Hu, Shenwen Du, Zhendong Lu, Jiming Jin, Chao Xu

**Affiliations:** ^1^ College of Resources and Environment Yangtze University Wuhan 430100 China; ^2^ Hubei Key Laboratory of Petroleum Geochemistry and Environment Yangtze University Wuhan 430100 China; ^3^ College of Water Resources and Architectural Engineering Northwest A&F University Yangling Shaanxi 712100 China; ^4^ School of Resource and Environmental Science Wuhan University Wuhan Hubei 430079 China; ^5^ Interdisciplinary Graduate Program in Informatics The University of Iowa Iowa City IA 52242 USA; ^6^ College of Resource and Environment Xinjiang Agricultural University Urumqi 830052 China; ^7^ Xinjiang Key Laboratory of Soil and Plant Ecological Processes Urumqi 830052 China

**Keywords:** air pollution, exposure risk, global, SDGs, WHO's new AQG

## Abstract

To reduce the high burden of disease caused by air pollution, the World Health Organization (WHO) released new Air Quality Guidelines (AQG) on September 22, 2021. In this study, the daily fine particulate matter (PM_2.5_) and surface ozone (O_3_) data of 618 cities around the world is collected from 2019 to 2022. Based on the new AQG, the number of attainment days for daily average concentrations of PM_2.5_ (≤ 15 µg m^−3^) and O_3_ (≤ 100 µg m^−3^) is approximately 10% and 90%, respectively. China and India exhibit a decreasing trend in the number of highly polluted days (> 75 µg m^−3^) for PM. Every year over 68% and 27% of cities in the world are exposed to harmful PM_2.5_ (> 35 µg m^−3^) and O_3_ (> 100 µg m^−3^) pollution, respectively. Combined with the United Nations Sustainable Development Goals (SDGs), it is found that more than 35% of the world's cities face PM_2.5_‐O_3_ compound pollution. Furthermore, the exposure risks in these cities (China, India, etc.) are mainly categorized as “High Risk”, “Risk”, and “Stabilization”. In contrast, economically developed cities are mainly categorized as “High Safety”, “Safety”, and “Deep Stabilization.” These findings indicate that global implementation of the WHO's new AQG will minimize the inequitable exposure risk from air pollution.

## Graphical abstract

Only 10% of days met PM_2.5_ AQG Target (15 µg m^−3^), while 90% met O_3_ AQG Target (100 µg m^−3^). Global decrease in cities with PM_2.5_‐O_3_ compound pollution, from 43.9% in 2019 to 36.8% in 2022; Over 60% of cities at Stabilization risk for PM_2.5_, and 20% for O_3_.

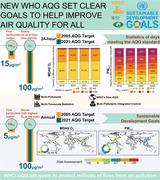

## 1 Introduction

With global climate change, air pollution has become one of the most serious environmental threats to human health.^[^ ^1^ ^]^ Evidences have proved that severe air pollution has direct adverse effects on human health,^[^ ^2^, ^3^ ^]^ ecology,^[^ ^4^ ^]^ and socioeconomics.^[^ ^5^ ^]^ Air pollution can lead to 8.8 million deaths and more than $200 billion loss in the economy worldwide each year, according to recent studies.^[^ ^6^, ^7^ ^]^ In 2005, the WHO revised the AQG, which was originally published in 1987, with a focus on health protection.^[^ ^8^ ^]^ By contrast to the AQG of 1987, the revised AQG of 2005 updated a multitude of guideline values for air pollutants, such as PM_2.5_ and O_3_. And it also offered the referable interim targets for air quality management in various countries worldwide by establishing phased targets.^[^ ^1^ ^]^

The 2005 edition of the AQG provides an important theoretical basis for facilitating a comprehensive understanding of global air pollution and for the in‐depth studies about crucial concerns (e.g., the effects of air pollution on human health). Simultaneously, it also offers citations for legislation, strategies, and planning, especially for the formulation of standard to reduce air pollution levels and alleviate the related health burden in different countries.^[^ ^9^ ^]^

It is an indisputable fact that after implementing mandatory policies to improve air quality, countries like China and South Korea have seen a significant decrease in air pollution incidents.^[^ ^10^, ^11^ ^]^ Conversely, Over the past three decades, air quality in regions like the United States and Europe has consistently met satisfactory standard.^[^ ^12^, ^13^ ^]^ However, air quality management policies introduced locally have not shown significant results.^[^ ^1^ ^]^ In addition, a growing evidence base suggests that air pollutants can cause significant harm to human health even at concentrations below those considered safe.^[^ ^14^, ^15^ ^]^ In light of above conflicting perspectives, there is an urgent need for a new air quality assessment system that can reassess and redefine the current state of global air quality in the contemporary era. The WHO has formulated a new AQG set in 2021, based on a comprehensive review of contemporary environmental health research and incorporating considerations of uncertainty.

The new AQG is deemed to be capable of safeguarding the well‐being of populace at present.^[^ ^8^ ^]^ At the same time, it makes a positive contribution to achieving the UN 2030 SDGs 11 (sustainable cities and communities), and 13 (climate action).^[^ ^16^, ^17^, ^18^ ^]^ In the forthcoming times, challenge of combating the air pollution with climate change will be imperative. The newly established AQG will serve as a crucial tool to mitigate air pollution and climate change.^[^ ^19^ ^]^ Actually, the distribution disparity of air pollution around the world is becoming larger,^[^ ^20^ ^]^ particularly in low‐income and middle‐income countries. In these countries, rapid urbanization and economic growth depend primarily on the burning of fossil fuels, which further increases air pollution. It also suggests that the most vulnerable and marginalized groups with lower socioeconomic status are at a higher risk of exposure to air pollution and bear a heavier burden of disease.^[^ ^21^, ^22^ ^]^ The unequal burden of disease caused by air pollution will be exacerbated with the advancement of socio‐economic development.

Therefore, the exploration of air pollutant concentrations changes in different regions around the world is of crucial importance in the context of the new version of AQG. Over the course of the last three decades, numerous scholars have carried out comprehensive examinations and investigations of atmospheric pollutant levels at a global, continental, national, and urban scale, by utilizing various modeling methodologies and employing the 1987 and 2005 AQG as benchmarks.^[^ ^23^, ^24^ ^]^ Nonetheless, there is still a lack of research on the characteristics of global atmospheric pollutant concentrations changes based on the new AQG. Thus, the aim of this study was to assess the relative changes of PM_2.5_ and O_3_ concentrations and spatiotemporal distribution of air pollution in cities worldwide when implementing the new WHO AQG target globally. In addition, the compound pollution and exposure risks under the SDGs were also explored.

In this study, the PM_2.5_ and O_3_ daily average concentrations data for 618 cities from 1 January 2019 to 31 December 2022 will be used to: 1. Exploring the temporal and spatial patterns, seasonal changes and exposure risk of global PM_2.5_ and O_3_ concentrations under the new AQG, through spatial analysis and risk assessment and other methods and models; 2. Assessing sustainability pathways under the new context of the AQG. In summary, under the auspices of the United Nations 2030 Agenda for Sustainable Development, examining the spatiotemporal distribution characteristics of PM_2.5_ and O_3_ pollution and categorizing exposure risk levels for assessment can aid in identifying regions at high pollution risk. This approach provides a foundation for the formulation of effective intervention measures aimed at reducing health risks in public areas. In addition, it fosters the dual objectives of environmental protection and public health enhancement, thereby contributing to the achievement of the United Nations SDGs for 2030. In fact, there is growing evidence that co‐management of PM_2.5_ and O_3_ is an important strategy for future air quality management. While this perspective is not commonly emphasized in existing research, it is crucial for achieving global environmental and public health objectives. Our study employs the latest AQG to delineate areas of combined pollution. This approach facilitates a clearer identification of critical regions affected by compound pollution, thereby enabling the development of more tailored atmospheric management strategies. Such results provide a scientific basis for future to achieve multifaceted governance goals in the global Air‐Climate‐Health sectors.

## 2 Experimental Section

### 2.1 Study Area

**Figure 1:**
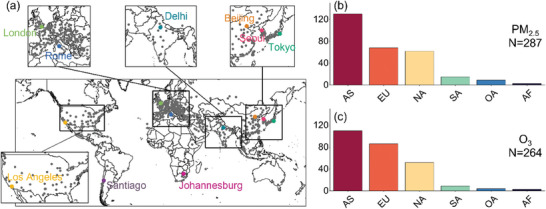
a) Spatial distribution of study urban sites. b,c) Number of urban sites with PM_2.5_ and O_3_ valid data for each continent.

For this investigation, the air quality data of 618 cities around the world was collected from 2019 to 2022. Based on the degree of differences in the spatial distribution of urban sites, the number of urban sites in different continents were collected. The findings indicate that Asia (AS) had the highest number of urban sites, with 130 (PM_2.5_) and 110 (O_3_), followed by Europe (EU), North America (NA), South America (SA), and Oceania (OA). Conversely, Africa (AF) had the lowest number of urban sites, with less than 10. Meanwhile, taking into account data completeness, air pollution levels, GDP levels, population density, industrial output, and other characteristics, nine typical representative cities were selected from different continents to explore the characteristics of PM_2.5_ and O_3_ concentrations changes under the new AQG target at the city scale. These cities were Beijing, China; Tokyo, Japan; Seoul, Korea; Delhi, India; London, United Kingdom; Rome, Italy; Johannesburg, South Africa; Los Angeles, United States; and Santiago, Chile (**Figure** **1**).

### 2.2 Data and Preprocessing

Daily site monitoring data for PM_2.5_ and O_3_ were acquired from the Air Quality Open Data Platform, spanning from January 1, 2019 to December 31, 2022 (https://aqicn.org/data‐platform/). To ensure the dependability of the monitoring data, the following data processing steps were executed: initially, the time series of monitoring data for each urban sites were assumed, mandating that the number of actual measured pollutant concentrations values per month should not be less than 27 (with the exception of February, where the daily average concentrations value should not be less than 25), and eliminated sites that did not meet the specified criteria. It should be noted that the daily average O_3_ concentrations were obtained by calculating the maximum 8‐h continuous average concentrations during the day and was denoted as “MDA8” (maximum of daily 8‐h moving average).^[^ ^25^ ^]^ In this study, O_3_ refers to MDA8 O_3_ unless otherwise noted. After above treatments, the quantity of municipalities satisfying the criteria outlined in this investigation amounted to 287 for PM_2.5_ and 264 for O_3_.

### 2.3 Indicators and Criteria

This article utilizes AQG established by the WHO in 2021 to identify threshold indicators for PM_2.5_ and O_3_. These indicators serve as the foundations for assessing the level of exposure risk to PM_2.5_ and O_3_ pollution. Zhu et al. provides an in‐depth exposition of the significant modifications and application extent of the 2021 Global AQG.^[^ ^8^ ^]^ **Table** 1 provides the details of PM_2.5_ and O_3_ concentrations thresholds employed in this investigation. It is noteworthy that the new AQG issued by the WHO do not establish a threshold criterion for the annual average concentrations of O_3_. The present study utilized the regulations promulgated by the United States Environmental Protection Agency in 2015 to ascertain whether the annual average concentrations of O_3_ surpassed the prescribed benchmarks. According to the US Environmental Protection Agency (2015),^[^ ^25^ ^]^ if the average maximum 8‐h concentrations of the fourth highest O_3_ in the year does not surpass the new AQG Target (100 µg m^−3^), it was deemed as an adherence to the standard.

**Table 1:** Interim targets for PM_2.5_ and O_3_ in the 2021 edition of the Global AQG.

Pollutant [µg m^−3^]	Averaging time	AQG [2021]				
		Interim Target [IT]				Target
		1	2	3	4	
PM_2.5_	Annual	35	25	15	10	5
	24‐h	75	50	37.5	25	15
O_3_	8‐h	160	120			100

To investigate the distribution characteristics of pollutants on different time scales under the requirements of the new AQG. Daily, monthly, seasonal, and yearly averages of PM_2.5_ and O_3_ concentrations, respectively, were computed. In the new AQG, monthly average pollutant concentration thresholds are not explicitly given, so concentration thresholds were used from other time scales.^[^ ^26^, ^27^, ^28^, ^29^, ^30^ ^]^ The seasonal averages are provided in appendix (Figures S1–S4, Supporting Information), and the seasonal maximum of O_3_ (Figures S5 and S6, Supporting Information) was separately analyzed.

### 2.4 Trend Analysis of Exceedance Days

To investigate the trend of exceedance days in cities worldwide between 2019 and 2022, we utilized a trend analysis model to analyze the trend of exposure days within different thresholds.^[^ ^31^, ^32^ ^]^ The formula used is presented Equation (1):

(1) $$\begin{array}{c} Trend\;=\frac{n\sum_{i=1}^{n}(i\times Days_{i})-(\sum_{i=1}^{n}i)(\sum_{i=1}^{n}Days_{i})}{n\sum_{i=1}^{n}i^{2}-{\left(\sum_{i=1}^{n}i\right)}^{2}}\; \end{array}$$

where *Days* represents the number of days when pollutant concentrations exceeds certain thresholds in each city; *n* is the time span, which is 4 years in this study; *i* is the year of observation; and *Days_i_* represents the number of days when pollutant concentrations exceeds certain thresholds in the i‐th year. It is important to note that the same day is not counted twice in a row. For example, if a certain day's concentrations level meets both threshold 1 and threshold 2, the lower threshold was used as the decision criterion. A linear regression analysis was conducted on the time series data of the number of days exceeding thresholds, with time as the independent variable. The resulting slope reflects the trend in the number of exceedance days over time. If the variable changes smoothly, the slope is likely to be similar to the rate of change and can reflect the trend of that data set, with significantly positive values of the slope indicating an increasing trend in the number of exceedance days and significantly negative values of the slope reflecting the opposite trend.^[^ ^32^ ^]^

### 2.5 Criteria for Assessing PM_2.5_‐O_3_ Compound Pollution

Different types of air pollution have different effects on human health and ecology. To investigate the spatial variation of compound pollution, the study of Niu et al.^[^ ^33^ ^]^ was referred. For PM_2.5_, the PM_2.5_ annual concentration interim target 1 (35 µg m^−3^) was used. For O_3_, it has been shown that O_3_ concentrations are decreasing and that low concentrations of O_3_ can also have an impact on human health.^[^ ^33^, ^34^, ^35^, ^36^, ^37^, ^38^, ^39^ ^]^ Hence, we adopt the more stringent AQG Target (100 µg m^−3^) as the threshold for O_3_ concentration. The specific rules are followed in **Table** **2**.

**Table 2:** Classify the dominant types of pollution.

Standard	Classifications
ρ(PM_2.5_)>35 µg m^−3^ and ρ(O_3_)>100 µg m^−3^	PM_2.5_‐O_3_ compound pollution (P‐O)
ρ(PM_2.5_)≤35 µg m^−3^ and ρ(O_3_)>100 µg m^−3^	O_3_ dominant pollution
ρ(PM_2.5_)>35 µg m^−3^ and ρ(O_3_)≤100 µg m^−3^	PM_2.5_ dominant pollution
ρ(PM_2.5_)≤35 µg m^−3^ and ρ(O_3_)≤100 µg m^−3^	Clean

### 2.6 Assessment of Exposure Risks

The SDGs related to air pollution aims to reflect air quality and its impact on the health of urban populations. On this basis, to investigate the exposure risk levels of global cities under the new AQG requirements, ∖the study of Lim et al.^[^ ^40^ ^]^ was referred and categorize the exposure risk levels into the following six types: High Risk (HR), Risk (R), Stabilization (ST), Deep Stabilization (DST), Safety (S), and High Safety (HS). The specific classification criteria are shown in **Table** **3**.

**Table 3:** Classify the risk level of potential exposure.

PM_2.5_ Standard	O_3_ Standard	Trend	Classifications
ρ(PM_2.5_)≤25 µg m^−3^	ρ(O_3_)≤100 µg m^−3^	Decreasing	High safety
ρ(PM_2.5_)≤25 µg m^−3^	ρ(O_3_)≤100 µg m^−3^	Increasing	Safety
25 µg m^−3^<ρ(PM_2.5_)≤35 µg m^−3^	100 µg m^−3^<ρ(O_3_)≤120 µg m^−3^	Decreasing	Deep stabilization
35 µg m^−3^<ρ(PM_2.5_)	120 µg m^−3^<ρ(O_3_)	Decreasing	Stabilization
25 µg m^−3^<ρ(PM_2.5_)≤35 µg m^−3^	100 µg m^−3^<ρ(O_3_)≤120 µg m^−3^	Increasing	Risk
35 µg m^−3^<ρ(PM_2.5_)	120 µg m^−3^<ρ(O_3_)	Increasing	High risk

These thresholds were selected from the World Health Group's AQG. In addition, aided by the Mann Kendal method,^[^ ^41^ ^]^ pollutant concentrations were analyzed to judge whether they have increased or decreased during the last 4 years. Then were classified as Increasing and Decreasing Trends. The study results indicate that when applying the Mann–Kendall method to analyze PM_2.5_ data, 80.63% of the data were significant at the p<0.01 level, and 93.03% were significant at the p<0.05 level (Figure S7, Supporting Information). In the analysis of O_3_ data, 68.18% of the data were significant at the p<0.01 level, and 88.26% at the p<0.05 level (Figure S7, Supporting Information).

## 3 Results and Discussion

### 3.1 Statistics of Days Meeting the AQG Standard

#### 3.1.1 Statistics of Days Meeting the Standard of PM_2.5_ Concentrations

**Figure 2:**
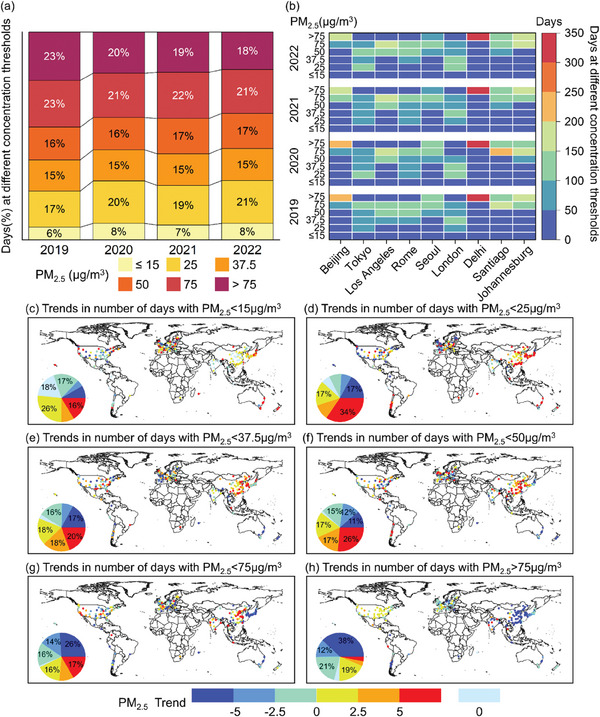
Spatial distribution of the cumulative number of days (a,b) exposed to different PM_2.5_ concentrations and the relative rate of change (c–h) for global cities and major cities.

**Figure** **2**a illustrates the proportion variation of cumulative days polluted by different PM_2.5_ concentrations in 287 cities worldwide from 2019 to 2022. Different concentrations are classified according to the new AQG criteria. Statistic results showed that the number of days when PM_2.5_ concentrations was below 15 µg m^−3^ (guideline value) in all cities accounted for less than 10% of the days in one year. Comparatively, the average number of days when cities were exposed to 15–75 µg m^−3^ was 250 days per year (accounting for approximately more than 70% of the days in one year) during the study period. Among these days, there were more than 70 days (accounting for 20% of the days in one year) when the cities were exposed to 50–75 µg m^−3^. Fortunately, with increasing PM_2.5_ concentrations, the cumulative number of days with exposure above 75 µg m^−3^ (IT1) had gradually decreased in all cities. That is, from 23% in 2019 to 20% in 2020 and finally to 18% in 2022. For the priority cities of concern, the sum of cumulative number of days with daily average concentrations below 15 µg m^−3^ (guideline value) was less than 40 days during the 4‐year study period. In contrast, the sum of the cumulative number of days with daily average concentrations above 75 µg m^−3^ (IT1) in the selected cities was over 800 days and was decreasing year by year.

Among them, Tokyo, Los Angeles, and London were exposed to > 75 µg m^−3^ (IT1) about less than 50 cumulative days per year. On the other hand, in Beijing and Delhi, the cumulative annual number of days exposed to more than 75 µg m^−3^ (IT1) had exceeded 200 days (Figure 2b). Figure 2c–h shows the trend and spatial distribution of the number of days within the specified PM_2.5_ concentrations thresholds. Overall, when PM_2.5_ concentrations are required to be less than 50 µg m^−3^ (Figure 2c–f), the number of cities with an increasing trend in the number of attainment days is greater than the number of cities with a decreasing trend in the number of attainment days. When PM_2.5_ concentrations are required to be greater than 50 µg m^−3^ (Figure 2g–h), the number of cities with a decreasing trend in the number of attainment days is approximately 1.8 times greater than the number of cities with an increasing trend in the number of attainment days. Specifically analyzed, when PM_2.5_ concentrations are required to be less than 15 µg m^−3^ (Figure 2c), 148 cities worldwide exhibit an increasing trend in attainment days, with the greatest increases being observed in Newcastle, Wollongong, and Sydney, with increasing trend values of 32.6, 50.3, and 55.3a^−1^, respectively. When PM_2.5_ concentrations are required to be less than 25, 37.5, and 50 µg m^−3^ (Figure 2d–f), the number of cities with significant growth trends (Trend > 5a^−1^) is 99, 56, and 76, respectively, which are mainly concentrated in the eastern part of China, Japan, and South Korea, such as Xiamen, Akita, and Busan. By contrast, 75 cities (< 75 µg m^−3^) and 109 cities (> 75 µg m^−3^) show more significant decreasing trends (Trend < −5a^−1^) when PM_2.5_ concentrations are required to be greater than 50 µg m^−3^ (Figure 2g–h), such as Sydney (−13.5 a^−1^), Lhasa (−16.6 a^−1^), and Sendai (−9.4 a^−1^).

#### 3.1.2 Statistics of Days Meeting the Standard of O_3_ Concentrations

**Figure 3:**
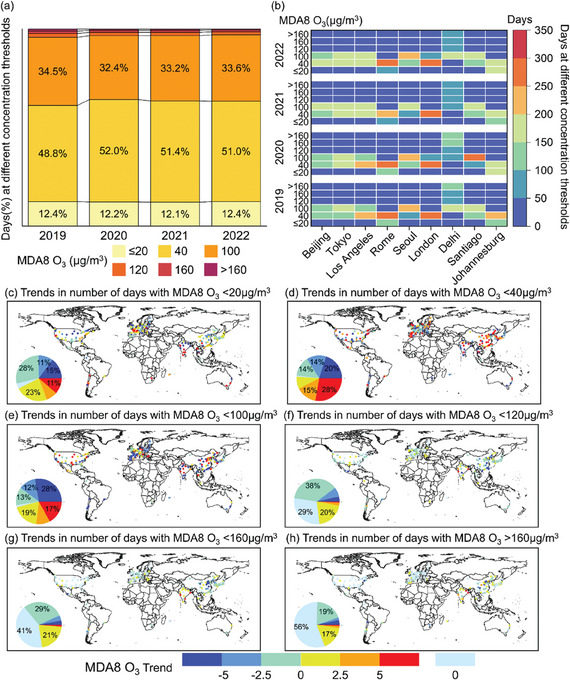
Spatial distribution of the cumulative number of days (a,b) exposed to different MDA8 O_3_ concentrations and the relative rate of change (c–h) for global cities and major cities.

**Figure** **3**a represents the percentage variation of cumulative days of the O_3_ daily average concentrations distribution in six intervals of 264 cities worldwide from 2019 to 2022. Our study showed that the cumulative number of days when O_3_ concentrations was above 100 µg m^−3^ (guideline value) accounts for no more than 4% of the year in all cities during the study period. And the cumulative number of days when O_3_ concentrations was below 40 µg m^−3^ can account for 60% of the year (more than 210 days). In addition, from the time‐varying perspective, the cumulative number of days exposed to less than 100 µg m^−3^ (guideline value) in all cities had increased every year, from 95.7% in 2019 to 96.7% in 2021 and further to 97% in 2022. The cumulative number of days with exposure below 100 µg m^−3^ during the study period exceeded 260 days (more than 71% of the annual days) in all major cities excluding Delhi, with Rome and Johannesburg having urban O_3_ concentrations below 40 µg m^−3^ for more than 300 days of the year.

It is noteworthy that the cumulative number of days exposed below 100 µg m^−3^ (guideline value) showed an annual increase from 268 days in 2019 to 288 days in 2020 and finally to 310 days in 2022. In comparison, Delhi was exposed to O_3_ concentrations above 100 µg m^−3^ for more than half of the days per year during the period studied (Figure 3b). Figure 3c–h shows the trend and spatial distribution of the number of days within the specified O_3_ concentrations thresholds. Overall, the number of cities with a significant increasing trend (Trend > 5a^−1^) in the number of attainment days when O_3_ concentrations are required to be less than 100 µg m^−3^ (Figure 3c–e) are 30, 75, and 45, respectively; In contrast, the number of cities with a significant downward trend (Trend < −5a^−1^) in the number of attainment days showed an upward trend of 39, 53, and 73, respectively. Spatially, cities in the eastern part of China and the southern part of Europe showing an increasing trend in the number of attainment days when the concentrations is less than 40 µg m^−3^ (Figure 3d), e.g. Beijing (13.7 a^−1^), Granada (23.9 a^−1^). When O_3_ concentrations is required to be greater than 100 µg m^−3^ (Figure 3f–h), it is interesting to note that the number of cities with no change in the number of attainment days shows an upward trend with increasing threshold, which is 77, 109 and 148, respectively. In terms of spatial distribution, these cities are concentrated in the United States and Europe, e.g. Boise and Rome. It also indicates that over 29% of cities observed no change in the number of high concentrations days under the new AQG. In contrast, some cities in China and Japan showed a decreasing trend in the number of attainment days, such as Changchun (−6 a^−1^) and Osaka (−2 a^−1^).

### 3.2 Annual and Seasonal Variation

#### 3.2.1 Annual Variation and Seasonal Variation of PM_2.5_

**Figure 4:**
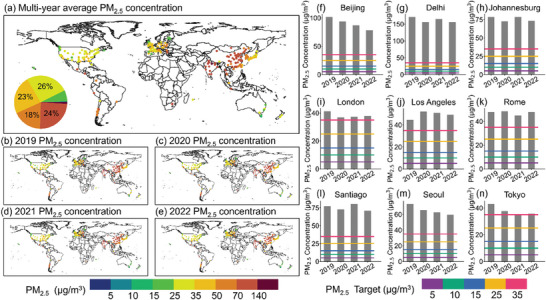
a) Spatial distribution of 4‐year average PM_2.5_ concentrations from 2019 to 2022; b–e) Spatial distribution of annual PM_2.5_ concentrations from 2019 to 2022; f–n) Annual PM_2.5_ concentrations in major cities.

The spatial distribution of 4‐year average PM_2.5_ concentrations in global cities from 2019 to 2022 is shown in **Figure** **4**a. It can be seen that approximately 33% of the world's cities were exposed to PM_2.5_ pollution with a concentration below 35 µg m^−3^. These cities are mainly located in the United States in North America and some other regions such as the United Kingdom, France, and Italy in Europe. In about 25% of cities, the 4‐year average PM_2.5_ concentrations there were above 70 µg m^−3^. These cities are mainly in China (93.78 µg m^−3^), East Asia and in India (120 µg m^−3^), South Asia. From a temporal perspective, the percentage of cities with average PM_2.5_ concentrations below 35 µg m^−3^ from 2019 to 2022 increased from 27% in 2019 to 31% in 2021 and further to 34% in 2022. These cities are mainly located in North America and parts of Europe, with concentrations ranging from 16.98 to 34.85 µg m^−3^. In contrast, the proportion of cities exposed to average PM_2.5_ concentrations above 70 µg m^−3^ decreased from 30% in 2019 to 25% in 2021 and further to 20% in 2022. The percentage of cities with average PM_2.5_ concentrations above 70 µg m^−3^ in 2022 was reduced by 10% in comparison with that in 2019. Cities with the most significant declines were mainly concentrated in East Asia, like China (15 observation stations) and South Korea (9 observation stations). For the major cities, annual PM_2.5_ concentrations in London, Los Angeles, Rome, and Tokyo had not exceeded 50 µg m^−3^ during the study period. In Beijing, Delhi, Johannesburg, and Santiago, the annual PM_2.5_ concentrations had exceeded 70 µg m^−3^. Fortunately, there were significant decreases in annual PM_2.5_ concentrations in Beijing (−22.9%), Seoul (−18.8%), and Tokyo (−16.9%). And in Delhi (−8.8%), Johannesburg (−6%), and Santiago (−8%), the decrease in PM_2.5_ concentrations was smaller. Furthermore, the annual PM_2.5_ concentrations in Los Angeles showed an inverted “V” pattern, and the peak reached in 2020 (52.3 µg m^−3^). Although the annual PM_2.5_ concentrations in Los Angeles did not exceed 70 µg m^−3^, it had increased by 10.1% from 2019 to 2022.

**Figure 5:**
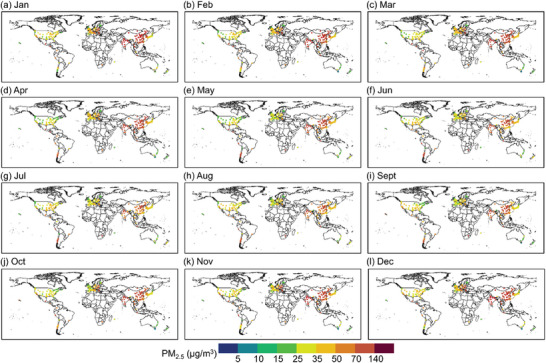
Spatial distribution of the monthly average concentrations of PM_2.5_ for the years 2019–2022.

Analysis of the seasonal variation (**Figure** **5**) showed that high levels of monthly average PM_2.5_ concentrations (> 35 µg m^−3^) occurred in cities during winter (Dec–Feb) and spring (Mar–May). The monthly average PM_2.5_ concentrations was relatively lower in summer (Jun–Aug) and autumn (Sept–Nov). Spatially, it showed a significantly high PM_2.5_ concentrations pollution area and two low PM_2.5_ concentrations pollution areas. The high‐pollution area was mainly concentrated in the Asian region, and the low‐pollution areas were mainly distributed in North America and southwestern Europe. Specifically, during the winter months (Dec–Feb), more than 65% of cities had a monthly average PM_2.5_ concentrations greater than 35 µg m^−3^, and more than 30% cities had a monthly average PM_2.5_ concentrations greater than 70 µg m^−3^. These cities (> 70 µg m^−3^) were mainly located in China (51 observation stations), India (14 observation stations) in South Asia, and some countries in Europe (9 observation stations). The PM_2.5_ pollution was worst in January and February. More than 5% of the world's cities had average PM_2.5_ concentrations above 140 µg m^−3^. In the worst polluted countries, there were 16 and 11 cities with concentrations above 140 µg m^−3^ in China and India, respectively. The spatial variation characteristics of global PM_2.5_ concentrations in the spring months were similar to that of PM_2.5_ concentrations in the winter months. However, the number of cities exposed to 140 µg m^−3^ had decreased significantly to lower than 1%. Global PM_2.5_ concentrations had improved significantly in the summer. 37.6%, 39%, and 36.5% of cities worldwide in summer had concentrations below 35 µg m^−3^, with an increase of 34.7%, 37.8%, and 17%, respectively, compared with winter. These cities were mainly located in the United States, North America, and some European countries. For the hotspots of PM_2.5_ pollution (i.e., China and India), although the number of cities exposed to less than 35 µg m^−3^ had increased (11 observation stations), most parts of the cities (36 observation stations) were still exposed to 50–70 µg m^−3^. The PM_2.5_ change trend in autumn and summer was similar, but there was a notable difference that the number of cities globally exposed to 70–140 µg m^−3^ in autumn had increased significantly (more than 15%), with an increase of about 2% compared with summer. In addition, cities in Asia with PM_2.5_ concentrations exceeding 70 µg m^−3^ accounted for 88.78% (Winter), 87.34% (Spring), 67.65% (Summer), and 90.77% (Autumn) of the number of cities with PM_2.5_ concentrations exceeding 70 µg m^−3^ in the world, respectively (Figure S2, Supporting Information).

#### 3.2.2 Annual Variation and Seasonal Variation of O_3_

**Figure 6:**
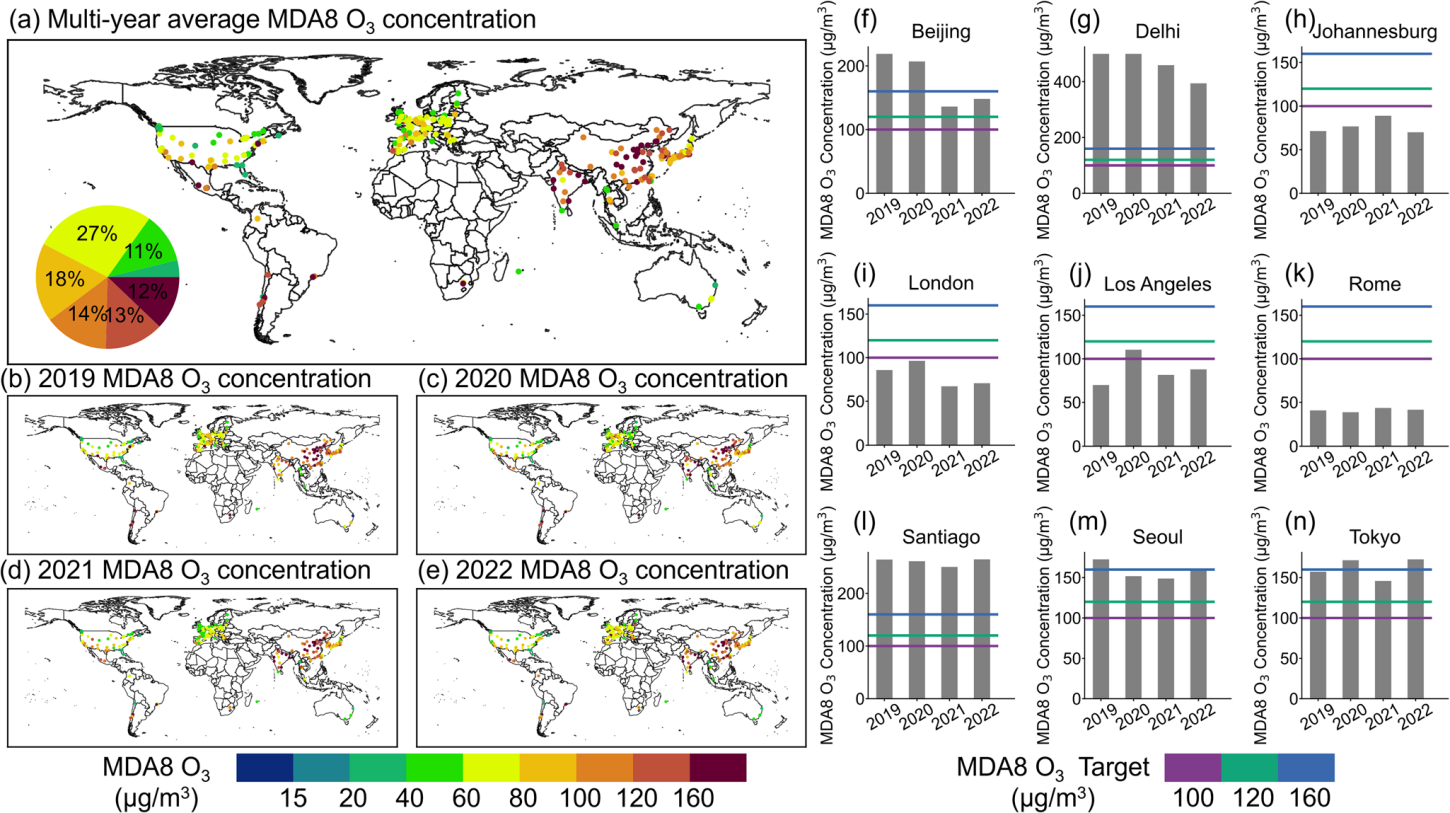
a) Spatial distribution of 4‐year average MDA8 O_3_ concentrations from 2019 to 2022; b–e) Spatial distribution of annual MDA8 O_3_ concentrations from 2019 to 2022; f–n) Annual O_3_ concentrations in major cities.

**Figure** **6** showed the spatial and temporal distribution of 4‐year average O_3_ concentrations in cities around the world between 2019 and 2022. It can be clearly seen that the concentrations of O_3_ showed a significant spatial variation and a strong temporal dynamic. In terms of the 4‐year average, the global 4‐year average O_3_ concentrations was 104.6 µg m^−3^, and the high‐value areas of global O_3_ pollution were mainly located in Nanning (112.6 µg m^−3^), and Changsha (139.1 µg m^−3^) in China, Lucknow (152.2 µg m^−3^) in India. Global O_3_ low pollution areas were concentrated in North American and European cities with 4‐year average O_3_ concentrations below 100 µg m^−3^. The characteristics of the annual spatial variation of O_3_ concentrations were similar to those of the 4‐year average spatial distribution of O_3_ concentrations. Specifically, about 58% of cities worldwide were exposed to concentrations below 100 µg m^−3^. These cities were mainly located in the United States and in most national areas from Europe. By comparison, about 26% of cities from East and South Asia had O_3_ concentrations above 100 µg m^−3^, and 12% of cities had concentrations above 160 µg m^−3^ (IT1).

During the study period, the number of cities worldwide exposed to O_3_ concentrations above 100 µg m^−3^ kept decreasing annually, from 42% in 2019 to 37% in 2020 and further to 34% in 2022. O_3_ concentrations in most cities in North America and Europe were below 100 µg m^−3^ from 2019 to 2022. For cities in China, Korea, and India, were chronically exposed to O_3_ concentrations above 100 µg m^−3^. For the selected key cities, O_3_ concentrations fluctuated from 2019 to 2022. In Beijing and Delhi, it showed a significant decreasing trend in O_3_ concentrations, but the annual O_3_ concentrations there exceeded 100 µg m^−3^, which were significantly higher than those in the other seven countries. Similarly, the annual O_3_ in Santiago, Seoul, and Tokyo were also at high pollution concentrations. But instead of a significant decreasing trend over time, the O_3_ concentrations in such cities showed an approximate “V” shaped trend (i.e., both decreasing and increasing). Conversely, the annual O_3_ concentrations in Johannesburg, London, Los Angeles, and Rome were close to 100 µg m^−3^ with the fluctuation change.

**Figure 7:**
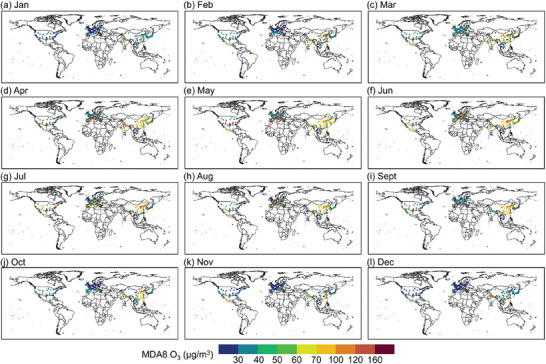
Spatial distribution of the monthly average concentrations of MDA8 O_3_ for the years 2019–2022.

**Figure** **7** shows the spatial distribution of monthly average O_3_ concentrations in cities in 2019 and 2022. We found that on average, more than 70% of cities worldwide had average O_3_ concentrations below 60 µg m^−3^ for all months, and less than 10% of cities were with average O_3_ concentrations above 100 µg m^−3^. With the change of seasons, the O_3_ concentration shows the change rule of high in spring and summer and low in autumn and winter. Global average O_3_ concentrations were 30.3, 32, and 37.9 µg m^−3^ during the winter months (Dec–Feb), respectively. On average, more than 47% of cities were exposed to average O_3_ concentrations below 40 µg m^−3^, and they were distributed in various regions around the world. Among these regions, there were more than 20% of cities located in European and North America with the average O_3_ concentrations below 30 µg m^−3^. During the spring, O_3_ concentrations in global cities began to gradually rise compared to that in the winter, and the number of cities exposed to average O_3_ concentrations below 40 µg m^−3^ decreased by more than 50% on average. On the other hand, about 20% cities were exposed to O_3_ concentrations between 60 and 100 µg m^−3^, which showed an increment of 50% in the number of cities compared with the winter time. The average O_3_ concentrations was further increased in the summer than in other seasons. Spatially, even though the average O_3_ concentrations remained below 40 µg m^−3^ in most cities worldwide, there was an increase in the number of cities with O_3_ concentrations above 60 µg m^−3^. About 13% cities, such as Phoenix (65.5 µg m^−3^) in the United States, Changsha (71.5 µg m^−3^) in China, and Kolkata (79.7 µg m^−3^) in India, had average O_3_ concentrations above 60 µg m^−3^ in June–August. During the autumn months, more than 65% of the world's cities were exposed to O_3_ concentrations below 40 µg m^−3^, which were mainly located in Japan, Korea, United States and most European countries. Strictly speaking, only about 10% of the world's cities had met an average monthly O_3_ concentrations of below 30 µg m^−3^ for year, and these cities were mainly located in the United States and Canada in the Americas. Furthermore, it was observed that the proportion of cities with quarterly maximum concentrations of O_3_ exceeding 100 µg m^−3^ decreased from 24.62% in summer to 17.8% in autumn and finally to 11.74% in winter (Figures S5 and S6, Supporting Information).

### 3.3 Spatial and Temporal Characteristics of PM_2.5_‐O_3_ Compound Pollution

**Figure 8:**
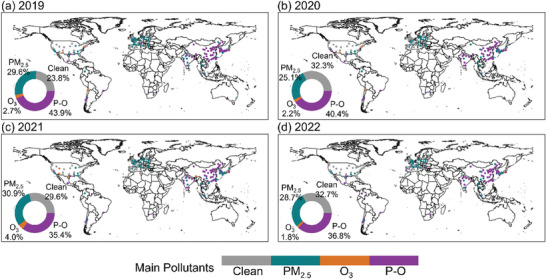
Spatial distribution of PM_2.5_ dominant pollution, O_3_ dominant pollution and PM_2.5_‐O_3_ composite pollution from 2019 to 2022.

From the new AQG perspective, we found that the global air pollution status during the study period can be clearly divided into the PM_2.5_ dominant pollution regions (European and American regions) and the PM_2.5_‐O_3_ compound pollution regions (Chinese and Indian regions) (**Figure** **8**). The statistical results showed that the number of cities with compound pollution (P‐O) accounted for more than 35% of the total number of cities worldwide during the study period, followed by clean cities (29.6%) and cities with PM_2.5_ dominant pollution (28.6%). In addition, the proportion of cities where O_3_ is the main pollutant did not exceed 4.0%. In terms of spatial and temporal variation, the number of cities with PM_2.5_ dominant pollution decreased from 29.6% in 2019 to 25.1% in 2020 worldwide, and the main decreasing regions in Europe were UK, France and Germany. However, the number of cities with PM_2.5_ dominant pollution had increased again in 2021, with an increment of 5.8% compared with 2020, and these areas were mostly located in the UK, Europe.

The proportion of global cities with PM_2.5_ dominant pollution decreased from 30.9% in 2021 to 28.7% in 2022. The spatial and temporal variation of global cities with O_3_ dominant pollution was similar to that of PM_2.5_ dominant pollution. More than 2% of cities around the world suffered from O_3_ dominant pollution from 2019 to 2020, with prominent regions like Queens, United States (101.5 µg m^−3^, 2020) and Calama, Chile (234 µg m^−3^, 2019). Compared with 2020, there was a 1.8% increase in the number of cities with O_3_ dominant pollution in 2021, and these cities with sudden deterioration in air quality were mainly distributed in the United States, such as San Antonio (114.3 µg m^−3^, 2021), Salt Lake City (120.3 µg m^−3^, 2021), and Phoenix (100.5 µg m^−3^, 2021). But in 2022, the number of cities with O_3_ dominant pollution had dropped. Notably, the PM_2.5_‐O_3_ compound pollution events happened frequently worldwide, but the number of cities with compound pollution decreased year by year, which ranged from 43.9% in 2019 to 36.8% in 2022. Cities suffering from PM_2.5_‐O_3_ compound pollution were mainly located in China (43 observation stations), Japan (8 observation stations), South Korea (19 observation stations), and India (12 observation stations). But the number of these cities had decreased from 84.2% in 2019 to 76.2% in 2020 and finally to 69% in 2022 (N = 101).

### 3.4 Exposure Risk Assessment

**Figure 9:**
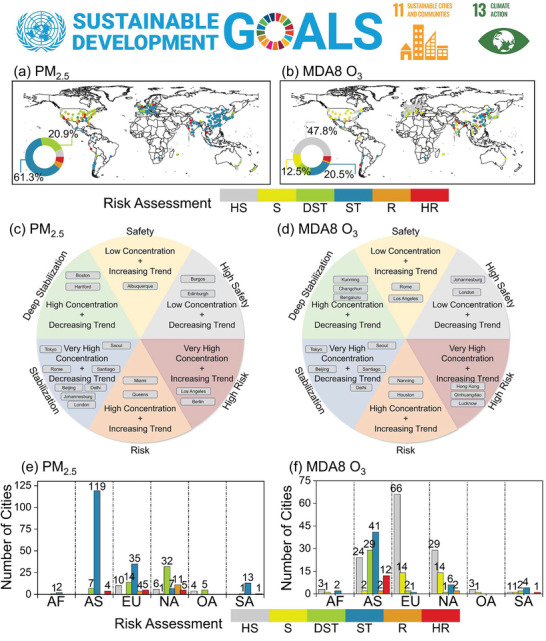
The spatial distribution of potential exposure risks (a,b). Potential exposure risks in focus cities (c,d). Number of cities with different potential exposure risks on each continent (e,f).

In **Figure** **9**, we analyzed the global exposure risk of PM_2.5_ and O_3_ pollution from 2019 to 2022 from the perspective of the SDGs. For PM_2.5_ (Figure 9a), more than 60% of the global cities had the exposure risk level of ST during the study period, and they are mainly concentrated in East Asia, South Asia, and Eastern European countries. For example, in China, India, and Poland, where the cities were characterized by very high concentrations and decreasing trends. About 20% of cities were at DST levels, which were mainly located in most of the United States. Unlike the ST type, these regions had high PM_2.5_ concentrations and decreasing trends. In addition, cities such as Indianapolis in the United States, Gdańsk in Poland, and Chandigarh in India had PM_2.5_ exposure levels at HR, accounting for about 5% of the total number of study cities. In contrast, only about 20% of cities worldwide had O_3_ exposure risk at the ST level during the study period (Figure 9b), which was mainly eastern Chinese cities and some Indian cities. 47.8% of cities had O_3_ exposure risk levels at HS. They are mostly located in continental Europe, which had low O_3_ concentrations and decreasing trends. And most United States cities had O_3_ exposure risk levels predominantly at S, with low O_3_ concentrations and increasing trends. In addition, Qinhuangdao in China and Mumbai in India had O_3_ exposure risk levels at HR. For a number of select cities (Figure 9c,d), our findings indicate that Tokyo, Seoul, Beijing, Santiago, and Delhi possess an exposure risk level of ST for both PM_2.5_ and O_3_. Meanwhile, London and Johannesburg possess an exposure risk level of ST and HS for PM_2.5_ and O_3_, respectively. Exposure risk level for PM_2.5_ and O_3_ in Rome is ST and S. And the exposure risk level for PM_2.5_ and O_3_ in Los Angeles is HR and S.

Figure 9e,f shows the number of cities at different exposure levels by continent. For PM_2.5_, the number of cities with an exposure risk level of ST in AS and EU accounted for more than 90% and 50%, respectively, and about 51.6% of the cities in NA had an exposure risk level of DST, which indicates that PM_2.5_ concentrations in these cities showed a decreasing trend during the study period. In addition, our study revealed that DST is the lowest exposure risk level for AF, AS, and SA, while HS is the lowest exposure risk level for EU, NA, and OA. Compared to other risk levels, the number of cities with an HS exposure risk level is no more than 10. Cities in the EU and NA with an exposure risk level of HS are the most common for O_3_, with 79.5% and 55.7% respectively. In AS, the highest number of cities with an exposure risk level in ST with 37.3%, followed by DST with 26.4%. In addition, there are 2 and 12 cities in AS with exposure risk classes R and HR, respectively, where O_3_ concentrations exceed 120 µg m^−3^ and show an increasing trend.

## 4 Discussion

### 4.1 PM_2.5_ and O_3_ Pollution

In this study, we found that global PM_2.5_ concentrations decrease from 57.44 µg m^−3^ in 2019 to 53.39 µg m^−3^ in 2021 and finally to 51.05 µg m^−3^ in 2022, but this is still more than ten times higher than the WHO 2021 annual average PM_2.5_ guideline (5 µg m^−3^). The highest PM_2.5_ annual average concentrations are observed in Asia, particularly in India (>120 µg m^−3^), where PM_2.5_ emissions from transport, industrial production and biomass combustion contribute to some extent to the local urban health burden.^[^ ^42^, ^43^, ^44^ ^]^ The primary factors influencing PM_2.5_ pollution in India include fossil fuel combustion, wood burning, urban industrial activities, and regional transport of pollutants.^[^ ^45^, ^46^, ^47^ ^]^ These factors lead to heightened levels of black carbon and polycyclic aromatic hydrocarbons in PM_2.5_, which cause substantial health hazard to the population, particularly in urban localities.^[^ ^45^, ^47^ ^]^ Due to the strict management of air quality in recent years and widespread closures due to global epidemics, the number of days with PM_2.5_ concentrations higher than 75 µg m^−3^ in China showed a significant decreasing trend, and the concentrations of PM_2.5_ in many cities showed a decreasing trend during the study period, but still far exceeded the PM_2.5_ target (5 µg m^−3^) set by the WHO for 2021.^[^ ^48^, ^49^, ^50^, ^51^ ^]^ Comparatively, Japan has a lower level of pollution with a PM_2.5_ concentrations of 38.06 µg m^−3^. This is closely associated with the local economic development level and energy consumption pattern; China is a country whose economy is still developing, while Japan is a developed country with a leading economic level. China's substantial energy consumption during its development has resulted in severe pollution.^[^ ^52^ ^]^ Additionally, the traditional energy sources relied upon by the local population will likely result in greater exposure to PM_2.5_ pollution compared to high‐income countries.^[^ ^53^ ^]^ While PM_2.5_ concentrations are lower in North America (32.74 µg m^−3^) and Europe (38 µg m^−3^), this can be attributed to local emission control policies in those regions.^[^ ^54^ ^]^ In addition, air quality control measures were initiated in Europe and the United States at an early stage. Subsequently, relevant government agencies implemented mandatory, targeted, and thorough air quality control policies to curb air pollution, which has further reduced PM_2.5_ pollution in the region.^[^ ^55^, ^56^, ^57^, ^58^ ^]^ In contrast, North America's urbanization rate is relatively high, and urban development is already mature, resulting in more comprehensive and efficient daily life and environmental management. On the other hand, China, as a developing country, experiences a significantly higher impact of urbanization rates on PM_2.5_ compared to North America.^[^ ^59^ ^]^ Regarding Europe, the invasion of Sahara dust is a major contributor to PM_2.5_ concentrations in the southern region, where the gathering of local pollutants is worsened by the transportation of external particles.^[^ ^60^ ^]^ Regarding the examination of monthly average levels, it is discovered that PM_2.5_ contamination is prevalent during winter months in China. Moreover, the number of cities with high pollution levels shows a decreasing trend from winter to summer, which is consistent with the results obtained by Zhao et al.^[^ ^48^ ^]^ During the winter months, cities in China heavily rely on coal and fossil fuels for heating. The combination of this practice and the impact of rainfall results in a higher concentrations of PM_2.5_ during winter compared to summer.^[^ ^61^ ^]^

During the analysis of O_3_ concentrations, we found that the annual O_3_ concentrations during the study period were 113.79, 104.31, 98.92, and 101.39 µg m^−3^, respectively. Similar to the results of the previous studies, we found that the O_3_ concentrations in the U.S. showed a decreasing trend during the study period.^[^ ^62^, ^63^, ^64^ ^]^ Lefohn et al., Zhang et al., and Goldberg et al. research points not only to stringent regulations on vehicle emissions, fume treatment, and solvent storage, but also to U.S. cleanup initiatives for power plant fuels as an important reason for NOx and VOCs reductions, which in turn have improved local O_3_ pollution.^[^ ^43^, ^65^, ^66^ ^]^ In addition, we observed low O_3_ concentrations in Australia (53.38 µg m^−3^).^[^ ^67^ ^]^ The research exhibits significantly O_3_ pollution in Asia, particularly in China and India, with the highest concentrations reaching 138 µg m^−3^, almost twice as high as those in the U.S. adhering to the latest AQG requirements. Despite a decreasing trend, Eastern China is the worst hit by O_3_ pollution, with concentrations exceeding the 2021 AQG standard (100 µg m^−3^).^[^ ^68^ ^]^ Seasonal differences in urban ozone concentrations are mainly influenced by temperature, and we observe that peaks in O_3_ concentrations occur during the summer months when temperatures are higher, due to the favorable conditions for O_3_ formation provided by intense solar radiation and rising temperatures.^[^ ^69^ ^]^ This effect is particularly pronounced in the cities of northern, eastern, and northeastern China. This finding aligns with the results of the He et al. study.^[^ ^70^ ^]^ In addition, our results show a significant increasing trend in O_3_ concentrations in London and Santiago in 2020. This may be due to the fact that local ozone concentrations are mainly influenced by NO and that during the outbreak there was a decrease in vehicle generated NO leading to an increase in O_3_ concentrations throughout the city.^[^ ^71^, ^72^, ^73^ ^]^ Additionally, research by Calatayud et al. indicates that vehicle electrification has enormous potential to reduce urban pollution in Europe.^[^ ^73^ ^]^ In summary, the formation of O_3_ pollution is a complex process influenced by various factors, such as meteorological conditions (including solar radiation),^[^ ^74^, ^75^ ^]^ human activities,^[^ ^76^ ^]^ precursor concentrations,^[^ ^64^ ^]^ and emission intensity.^[^ ^77^, ^78^ ^]^ These factors can have a significant impact on O_3_ concentrations. Therefore, the complex interactions between O_3_ concentrations and various drivers need to be further investigated. At the national level, the control of O_3_ is mainly reflected in two aspects, on the one hand, it is necessary to reduce the precursor emissions of O_3_ (NOx and VOCs) at the same time to reduce the occurrence of O_3_ pollution events; On the other hand, some anthropogenic measures (e.g., introduction of artificial rainfall during high summer temperatures) can also reduce O_3_ concentrations.

Our study indicates that China is the high‐risk area for compound pollution, with 86.05% of cities affected. The interaction between PM_2.5_ and O_3_ is intricate. Especially during wintertime, PM_2.5_ concentrations tend to be higher in cities located in Northern China, as a result of the heating season's effects. This may lead to a decrease in solar radiation, which may affect the photochemical production of O_3_.^[^ ^79^ ^]^ In cities located in the southern region, O_3_ creation may be influenced more by temperature and humidity than by PM_2.5_ concentrations because of the comparatively high levels of humidity and temperature and lower concentrations of PM_2.5_.^[^ ^74^, ^80^ ^]^ Second, the common precursors of NOx and VOCs can also influence the incidence of compound air pollution events. NOx and VOCs generate ozone via photochemical reactions under sunlight exposure. For PM_2.5_, NOx plays an important role in the formation of secondary inorganic aerosols (e.g., nitrates), while VOCs are a major source of secondary organic aerosols. Under specific meteorological conditions and in certain regions, the formation and concentrations levels of O_3_ and PM_2.5_ are determined by the ratio of emissions of NOx and VOCs.^[^ ^76^, ^77^, ^78^, ^79^, ^80^ ^]^ For instance, in areas with VOC restrictions, reducing NOx emissions may result in increased O_3_ concentrations and vice versa.^[^ ^81^, ^82^ ^]^ Additionally, Jiang et al. research indicates that the atmospheric oxidizing capacity's strength impacts the production of PM_2.5_ and O_3_ in both northern and southern cities.^[^ ^76^ ^]^ Increased atmospheric oxidizing capacity speeds up the transformation of NOx and VOCs into O_3_ and PM_2.5_ secondary pollutants. Increased atmospheric oxidizing capacity, especially during pollution events, can lead to increases in nitrate and secondary organic aerosols, while also promoting O_3_ formation.^[^ ^76^ ^]^ In addition, northern and southern cities have different industrial structures and energy use patterns, which can lead to differences in the chemical composition and optical properties of PM_2.5_, which in turn affect the way O_3_ is produced.^[^ ^83^, ^84^ ^]^

### 4.2 Policies and Recommendations

It is widely acknowledged that AQG was not equipped with legal compulsory. However, it can serve as a recognized standard and provide a valuable reference for decision makers in different countries when formulating legislation, strategies, policies and plans, and can thereby help to mitigate local air pollution and its associated health burdens. Stringent policies aimed at reducing air pollutants have led to notable improvements in air quality in several countries with persistent air pollution issues, like China and India.^[^ ^85^, ^86^ ^]^ In 2020, the total annual concentrations of PM_2.5_ at 337 national control monitoring stations in Chinese cities was 33 µg m^−3^, which was below the annual concentrations standard (35 µg m^−3^) for the first time (https://www.mee.gov.cn/xxgk2018/). Simultaneously, our findings demonstrated that PM_2.5_ and O_3_ concentrations exhibit a noteworthy decrease in over 60% of cities worldwide based on the thresholds of the new AQG, with varying degrees of reduction in the days of surpassing the limits. Due to the sustainable enhancement of worldwide air quality, the old AQG have lost their predominant and motivational influence on the majority of cities that have attained compliance with the standard (e.g., Bangalore, São Paulo, Shenzhen).^[^ ^87^ ^]^ Hence, the implementation of a new AQG for cities that meeting the AQG standard will not only benefit for improving the air quality, but also reduce health burdens. Conversely, for developing cities undergoing swift economic growth and endeavoring to enhance the general well‐being of their populace, it is imperative to undertake a comprehensive evaluation of the disparity between viable environmental quality benchmarks and the novel AQG according to the local circumstances. Additionally, scientific modifications must be made to ensure conformity with the new AQG.

Concurrently, an increasing number of studies have demonstrated that socio‐economically disadvantaged groups, such as children,^[^ ^88^ ^]^ women,^[^ ^89^ ^]^ the elderly,^[^ ^90^ ^]^ and those with underlying health issue, would bear a greater risk of exposure to air pollution. This may further exacerbate the existing inequality in the distribution of medical resources and corresponding economic burdens, ultimately diminish our capacity to combat atmospheric pollution.^[^ ^91^ ^]^ In the future, air pollution‐related events associated with rural urbanization, ageing populations and industrial change are expected to increase, which may exacerbate the vulnerability of certain groups to air pollution,^[^ ^46^, ^92^ ^]^ especially in developing countries. Therefore, from an environmental equity and justice perspective, the new AQG facilitates the understanding of the gaps in air quality and associated health burdens between developed and undeveloped countries that exist today. In addition, the new AQG can inform the design of future policy interventions to eliminate these gaps. In more than this, the new AQG offers sustainable development recommendations for creating a more equitable, just, and healthy world.

Furthermore, the release of the new AQG represents an important opportunity for countries to realize the synergistic benefits of air, climate, and health, and to maximize the public health protection. According to scholarly research, there exists a correlation between climate change and air pollution, resulting in a comprehensive impact on the well‐being of the general public.^[^ ^93^ ^]^ Moreover, it has been observed that air pollutants and greenhouse gas emissions have the characteristics of “same root and origin”.^[^ ^94^ ^]^ Therefore, if the new AQG is successfully implemented in various countries, it will not only provide an excellent opportunity to reduce global air pollution levels, but also actively mitigate climate warming through its emission reduction benefits. And that will significantly reduce the health burden caused by air pollution and climate change. On this basis, decision‐makers should consider it as a whole in order to maximize the health benefits.

## 5 Research Limitations and Prospects

In this study, we investigated the spatial and temporal characteristics and exposure risks of PM_2.5_, O_3_, and compound pollution at global and urban scales under the new AQG requirements. It provides practical guidance for understanding the global situation of compound pollution and formulating targeted prevention policies. However, this study has some shortcomings. However, there are parts of this study that could be improved. The data distribution is focused, with more extensive pollution data for North America, Europe, and Asia—areas of focus for our study. In contrast, there is relatively less data available for Africa, South America, and Oceania, which are not the focus of our research. Second, our study relied heavily on data from monitoring stations in specific cities, which may introduce some level of error when attempting to generalize to the entire region's pollution levels. Finally, our study solely examines the risk of air pollution exposure on the global population at varying concentrations, without analyzing the epidemiological health burden caused.

In future research, we aim to investigate the meteorological and social factors contributing to PM_2.5_‐O_3_ compound pollution in global cities under the new AQG standard. Using factor‐driven, attributed‐analysis, and numerical models of atmospheric chemistry, we will quantitatively reveal the drivers of compound pollution and its impact on human health at global and urban scales. This research will provide theoretical support for achieving a more sustainable and healthier living environment in accordance with the SDGs.

## 6 Conclusion

In this study, we analyzed the number of exceedance days, spatial and temporal patterns, and trend variations of PM_2.5_ and O_3_ concentrations in 618 cities worldwide from 2019 to 2020, based on the new AQG perspective. We also explored the global urban PM_2.5_ and O_3_ compound pollution status and the risk of population exposure due to PM_2.5_ and O_3_ pollution under the new AQG. The main findings are as follows:

1) During the study period, the number of attainment days when daily average PM_2.5_ concentrations were required to be below the AQG target (≤ 15 µg m^−3^) was 10% of the year. In contrast, when O_3_ concentrations are required to be below the AQG standard (≤ 100 µg m^−3^), the number of attainment days is 90% of the year. On average, O_3_ concentrations significantly impacting human health are present on over 40% of the days per year. Spatially, the areas showing an increasing trend in the number of days with high PM_2.5_ pollution (> 75 µg m^−3^) are concentrated in the eastern United States; While China and India have experienced a significant downward trend in the number of highly polluted PM_2.5_ days, the number of days exceeding the standard is still higher than other cities in the statistics.
2) The percentage of cities exposed to average PM_2.5_ concentrations below 35 µg m^−3^ during the study period increases from 27% in 2019 to 31% in 2021 and further to 34% in 2022, mainly in the North American and European country regions; In contrast, the share of cities with average PM_2.5_ concentrations above 70 µg m^−3^ during the study period decreases from 30% in 2019 to 25% in 2021 and further to 20% in 2022, mainly in East Asia in China (15 observation stations) and South Korea (9 observation stations). For O_3_, the global 4‐year average O_3_ concentrations is 104.6 µg m^−3^, with approximately 58% of cities exposed to concentrations below 100 µg m^−3^, mainly in the United States in the Americas and in most country regions in Europe; In comparison, about 26% of cities in eastern and southern Asia have O_3_ concentrations above 100 µg m^−3^, and 12% of cities have concentrations above 160 µg m^−3^ (IT1). During the study period, the number of cities globally exposed to O_3_ concentrations above 100 µg m^−3^ is decreasing annually, from 42% in 2019 to 37% in 2020 and further to 34% in 2022.
3) In the new AQG perspective, on average, more than 35% of cities have PM_2.5_‐O_3_ compound pollution problems each year, but the number of cities with compound pollution decreases from 43.9% in 2019 to 36.8% in 2022 in terms of temporal trends; among them, China (37 observation stations), Japan (9 observation stations), South Korea (19 observation stations), and India (12 observation stations) are PM_2.5_‐O_3_ compound pollution prone regions.
4) The exposure risk results show that more than 60% and 20% of cities worldwide are at ST for PM_2.5_ and O_3_ exposure risk levels, respectively, mainly in Eastern Europe, East Asia and South Asia. In addition, the majority of urban PM_2.5_ and O_3_ exposure risk levels in countries such as China and India are at the ST. From an economic development standpoint, the exposure risk levels of PM_2.5_ and O_3_ pollution in developing country cities are primarily ST, R, and HR. In contrast, the exposure risk levels of PM_2.5_ and O_3_ pollution in developed country cities are mainly HS, S, and DTS.

## Conflict of Interest

The authors declare no conflict of interest.

## Supporting information

Supporting Information

## Data Availability

The data that support the findings of this study are available from the corresponding author upon reasonable request.
