# Supplementary material for: Toward Better and Healthier Air Quality: Global PM2.5 and O3 Pollution Status and Risk Assessment Based on the New WHO Air Quality Guidelines for 2021
Source: Glob Chall. 2024 Mar 26;8(4):2300258. doi: 10.1002/gch2.202300258 (PMC11009431; doi:10.1002/gch2.202300258)
Supplement: Supplementary file 1 — Supporting Information [file GCH2-8-2300258-s001.pdf]

Global
Challenges
Open Access

Supporting Information

for *Global Challenges.*, DOI 10.1002/gch2.202300258

Toward Better and Healthier Air Quality: Global $PM_{2.5}$ and O_3 Pollution Status and Risk Assessment Based on the New WHO Air Quality Guidelines for 2021

Jianhua Liu, Chao He, Yajun Si, Bin Li, Qian Wu, Jinmian Ni, Yue Zhao, Qixin Hu, Shenwen Du, Zhendong Lu, Jiming Jin and Chao Xu*

Table S1. Number of days per year when the concentrations of $PM_{2.5}$ exceeds the threshold.

Year	Target	Beijing	Tokyo	Los Angeles	Rome	Seoul	London	Delhi	Santiago	Johannesburg
2019	≤15	0	9	0	0	2	0	0	0	0
	25	19	65	46	41	22	69	0	1	0
	37.5	21	70	60	55	11	134	1	9	9
	50	27	99	121	111	53	79	3	35	32
	75	66	111	132	137	149	56	18	177	147
	>75	232	11	6	21	128	27	343	143	177
2020	≤15	0	13	6	3	8	1	0	0	0
	25	23	112	27	72	21	107	0	1	0
	37.5	27	66	38	57	26	132	0	4	15
	50	34	95	102	89	58	59	3	34	55
	75	69	74	165	102	135	48	41	203	163
	>75	213	6	28	43	118	19	322	124	133
2021	≤15	2	25	1	0	3	1	0	0	0
	25	28	99	16	60	33	80	0	5	2
	37.5	18	71	36	63	25	127	0	9	16
	50	36	93	101	97	59	67	0	55	31
	75	100	55	172	103	139	61	23	146	130
	>75	160	1	18	21	85	8	321	129	165
2022	≤15	8	16	1	2	9	0	0	0	0
	25	43	111	12	45	30	103	0	9	1
	37.5	31	65	49	55	35	110	1	22	13
	50	46	107	119	109	66	86	6	97	45
	75	78	66	180	126	137	51	30	113	155
	>75	159	0	4	28	88	15	328	124	151

Table S2. Number of days per year when the concentrations of MDA8 O_3 exceeds the threshold.

Year	Target	Beijing	Tokyo	Los Angeles	Rome	Seoul	London	Delhi	Santiago	Johannesburg
2019	≤20	29	5	17	104	3	32	0	39	118
	40	110	148	224	257	84	297	4	133	240
	100	129	180	124	4	211	34	152	170	5
	120	38	22	0	0	38	0	53	3	1
	160	43	8	0	0	23	2	56	4	1
	＞160	16	2	0	0	6	0	100	16	0
2020	≤20	18	7	10	112	1	29	0	3	161
	40	133	178	201	251	107	264	0	69	194
	100	137	155	144	3	212	72	80	261	9
	120	41	14	10	0	25	1	65	8	0
	160	25	8	1	0	19	0	102	8	2

	＞160	12	4	0	0	2	0	119	17	0
2021	≤20	25	2	8	87	3	31	0	32	141
	40	132	151	165	246	92	264	0	120	189
	100	150	164	171	11	193	49	85	151	11
	120	24	18	0	0	31	0	73	8	1
	160	13	6	0	0	23	0	92	16	2
	＞160	0	3	0	0	2	0	94	17	0
2022	≤20	18	4	8	65	1	30	0	43	176
	40	156	171	182	292	107	284	5	104	171
	100	136	166	174	8	209	51	162	167	16
	120	30	12	0	0	34	0	56	14	1
	160	22	8	1	0	10	0	66	14	1
	＞160	3	4	0	0	4	0	76	23	0

Table S3. Statistics on the number of cities in each continent under the new AQG $PM_{2.5}$ ($\mu g/m^3$) Targets.

Year	Continent	<5	<10	<15	<25	<35	<50	<70	<140	>140
2019	Africa	0	0	0	0	1	0	0	2	0
	Asia	0	0	0	0	4	25	22	74	5
	Europe	1	0	0	6	12	40	8	1	0
	North America	0	0	0	9	37	13	1	2	0
	Oceania	0	0	1	1	5	2	0	0	0
	South America	0	0	0	0	1	0	9	5	0
2020	Africa	0	0	0	1	0	0	0	2	0
	Asia	0	0	0	0	7	29	33	58	3
	Europe	1	0	0	8	27	23	7	2	0
	North America	0	0	0	5	43	9	4	1	0
	Oceania	0	0	1	3	5	0	0	0	0
	South America	0	0	0	0	1	3	6	5	0
2021	Africa	0	0	0	1	0	0	1	1	0
	Asia	0	0	0	0	11	23	37	56	3
	Europe	1	0	0	9	17	32	7	2	0
	North America	0	0	0	7	36	14	3	2	0
	Oceania	0	0	2	7	0	0	0	0	0
	South America	0	0	0	0	1	2	5	7	0
2022	Africa	0	0	0	1	0	0	1	1	0
	Asia	0	0	0	1	11	24	42	49	3
	Europe	1	0	0	9	14	36	7	1	0
	North America	0	0	0	6	45	8	1	2	0
	Oceania	0	0	2	7	0	0	0	0	0
	South America	0	0	0	0	2	4	7	2	0

Table S4. Statistics on the number of cities in each continent under the new AQG MDA8 O_3 ($\mu g/m^3$) Targets.

Year	Continent	<15	<20	<40	<60	<80	<100	<120	<160	>160
2019	Africa	0	0	0	1	1	0	0	0	1
	Asia	0	0	0	5	7	11	36	25	26
	Europe	1	0	0	10	40	29	3	1	2
	North America	0	0	10	9	18	6	2	1	6
	Oceania	1	0	0	0	1	2	0	0	0
	South America	0	0	1	0	0	2	0	2	4
2020	Africa	0	0	0	1	1	0	0	0	1
	Asia	0	0	0	7	7	16	26	33	21
	Europe	0	0	2	22	36	22	3	0	1
	North America	0	0	9	9	18	8	5	2	1
	Oceania	0	0	1	0	3	0	0	0	0
	South America	0	0	1	0	1	1	0	3	3
2021	Africa	0	0	0	1	0	1	1	0	0
	Asia	0	0	1	5	7	22	23	32	20
	Europe	0	0	0	31	44	8	3	0	0
	North America	0	0	8	8	18	10	5	3	0
	Oceania	0	0	1	2	1	0	0	0	0
	South America	0	0	2	0	2	1	1	1	2
2022	Africa	0	0	0	1	1	0	1	0	0
	Asia	0	0	1	4	6	25	29	25	20
	Europe	0	0	0	8	53	20	1	3	1
	North America	0	0	9	10	16	12	2	0	3
	Oceania	0	0	1	3	0	0	0	0	0
	South America	0	0	2	0	0	2	2	0	3

Figure S1 displays the quarterly average concentration values of $PM_{2.5}$ throughout the investigation period. The figure displays a significant decreasing trend in the number of cities with $PM_{2.5}$ concentrations exceeding 70$\mu g/m^3$ from winter to summer, with the percentage of these cities decreasing from 34.15% in winter (d), to 27.53% in spring (a), and finally to 11.85% in summer (b). Specifically, Asia (**Figure S2**) are the regions that best highlight the seasonal variations that characterize $PM_{2.5}$. Cities in Asia with $PM_{2.5}$ concentrations exceeding 70$\mu g/m^3$ accounted for 88.78% (Winter), 87.34% (Spring), 67.65% (Summer), and 90.77% (Autumn) of the number of cities with $PM_{2.5}$ concentrations exceeding 70$\mu g/m^3$ in the world, respectively. And we find that $PM_{2.5}$ concentrations are generally higher in central and eastern cities of China than in the southern and coastal ones. As an extreme example, in winter, the $PM_{2.5}$ concentrations in Shijiazhuang (148.27$\mu g/m^3$) is almost twice as high as that in Haikou (64.8$\mu g/m^3$). In addition, both Mumbai and Delhi in India exhibit $PM_{2.5}$ concentrations exceeding 140$\mu g/m^3$ during winter, categorizing them as highly polluted regions. By comparison, we found that Japan has the lowest $PM_{2.5}$ concentrations (37.97$\mu g/m^3$) in Asia and is

closer to the average levels in Europe (37.99μg/m^3) and the United States (31.79μg/m^3). In Europe, the number of cities with $PM_{2.5}$ concentrations exceeding 35μg/m^3 was 77.94% (Winter), 75% (Spring), 30.88% (Summer), and 42.65% (Autumn), respectively, and the quarterly mean $PM_{2.5}$ concentrations was always less than 50μg/m^3. In America, the number of cities with $PM_{2.5}$ concentrations exceeding 35μg/m^3 was 35.42% (Winter), 14.58% (Spring), 39.58% (Summer), and 37.5% (Autumn), respectively.

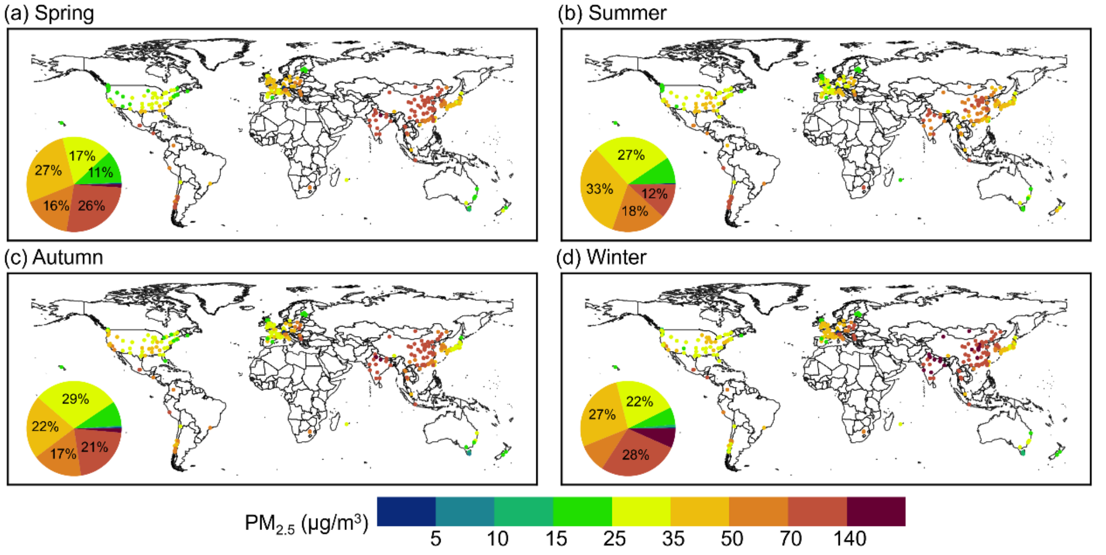

Figure S1. Spatial distribution of quarterly average $PM_{2.5}$ concentrations

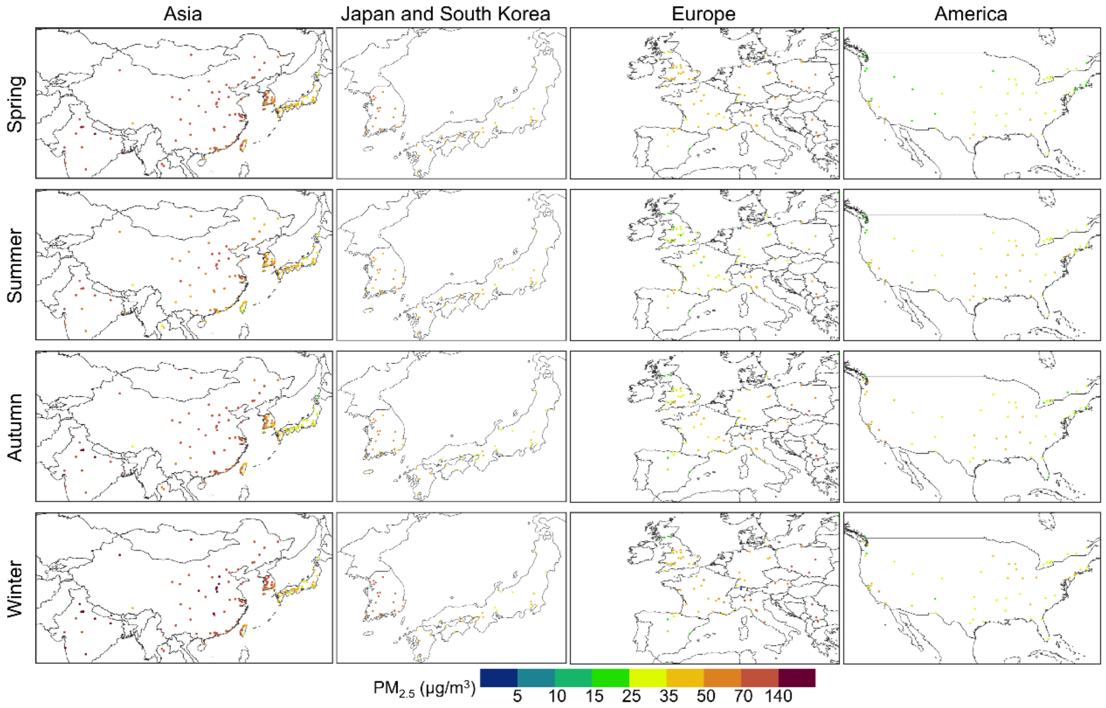

Figure S2. Spatial distribution of quarterly average $PM_{2.5}$ concentrations in Asia, Europe, and the United States.

Figure S3 displays the quarterly average concentration values of O_3 throughout the investigation period. The global average O_3 concentrations were 47.02μg/m^3 and 47.58μg/m^3 in spring and summer, respectively. Unlike $PM_{2.5}$, which displays seasonal variation, O_3 pollution is concentrated during the summer. The figures indicate that the percentage of the number of cities with O_3 concentration values below 40μg/m^3 decreased from 43.56% in spring (a), decreased to 34.84% in summer (b), and increased to 80.68% in winter (d), showing a decreasing and then increasing trend. And similar to $PM_{2.5}$, the area affected by O_3 pollution also spreads across central and eastern cities in China. Specifically, the average summer O_3 concentrations in China were 73.96μg/m^3 (**Figure S4**). Of the cities studied, 63.64% had average O_3 concentrations exceeding 60μg/m^3. As a result, China has the highest O_3 concentrations in the Asian region. Comparatively, Thailand and Japan had the lowest average O_3 concentrations in Asia, measuring at 19.35μg/m^3 and 50.57μg/m^3, respectively. From an urban perspective, during the summer in China, the cities of Shijiazhuang, Anyang, and Zhengzhou have the highest O_3 pollution, with concentrations of 142.02μg/m^3, 121.27μg/m^3, and 119.29μg/m^3, respectively. In addition, during the summer, average O_3 concentrations in Delhi, Taiyuan, and Jinan exceed 100μg/m^3. While the average O_3 concentrations in Europe and America were 33.09μg/m^3 and 38.93μg/m^3, respectively. Even in summer, only 1.16% of European cities and 18.92% of American cities had average O_3 concentrations that exceeded 60μg/m^3. Similarly, it was observed that the highest concentration of O_3 tends to happen during the summer months, as shown in **Figure S5**. Specifically, the percentage of cities with maximum O_3 concentrations exceeding 100μg/m^3 decreased from 24.62% in the summer, to 17.8% in the fall, and ultimately to 11.74% in the winter. In terms of spatial distribution (**Figure S6**), cities with high O3 pollution (maximum O_3 > 100μg/m^3) during summer are concentrated mainly in China and India. e.g. Shijiazhuang (253.83μg/m^3), Mumbai (261.85μg/m^3). During summer, high levels of O_3 pollution affect more than 50% of Chinese cities, while less than 10% of European and American cities with similar levels of O_3 pollution.

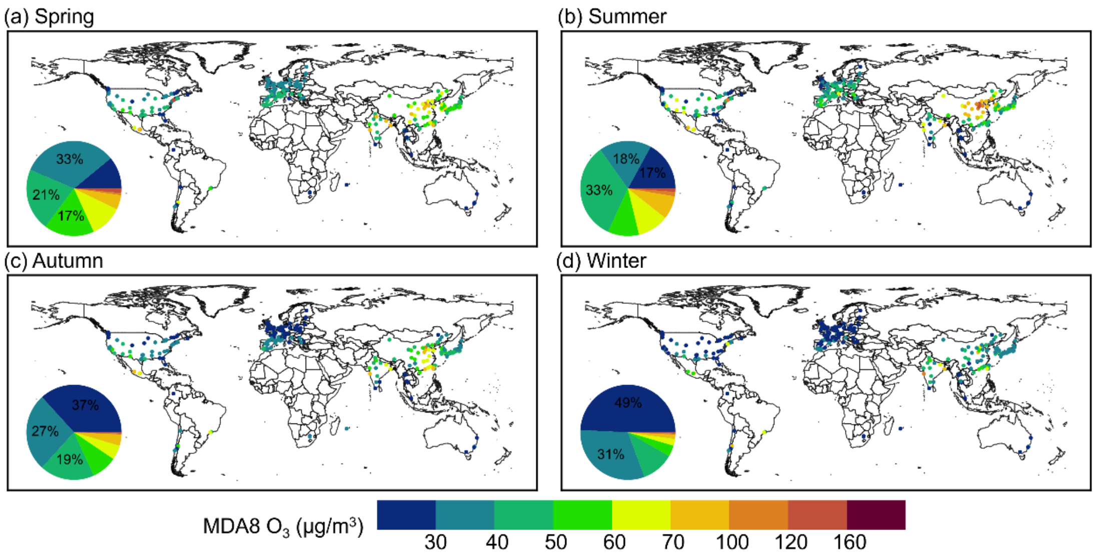

Figure S3. Spatial distribution of quarterly average MDA8 O_3 Concentrations

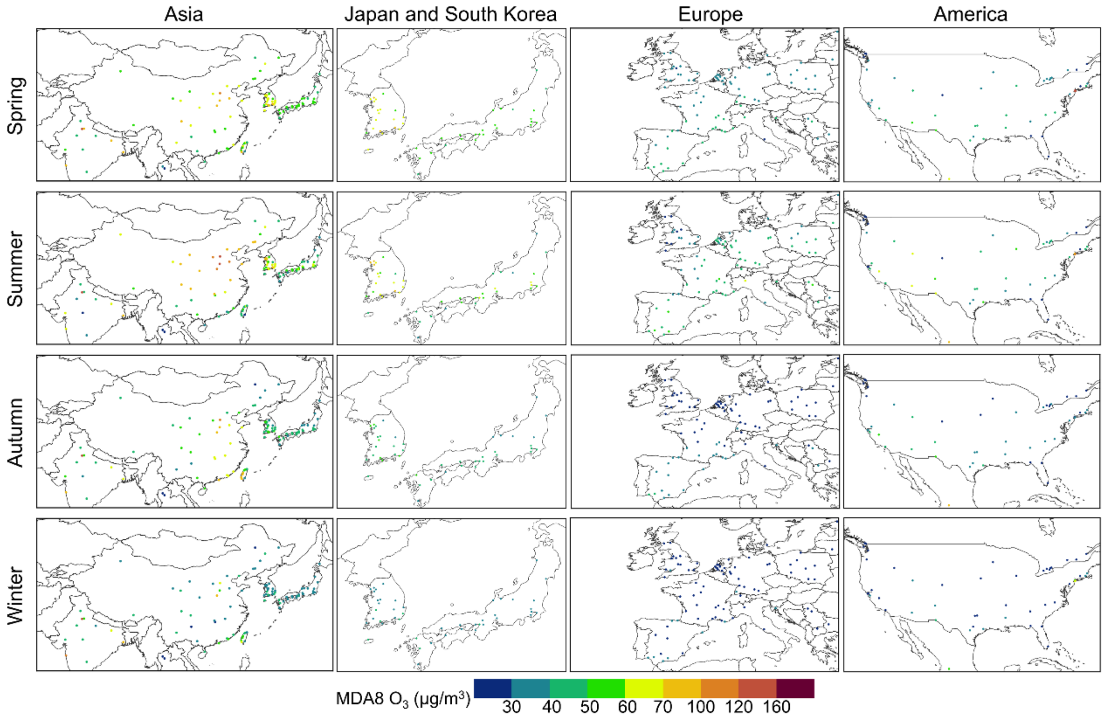

Figure S4. Spatial distribution of quarterly average MDA8 O_3 concentrations in Asia, India, Europe, and the United States.

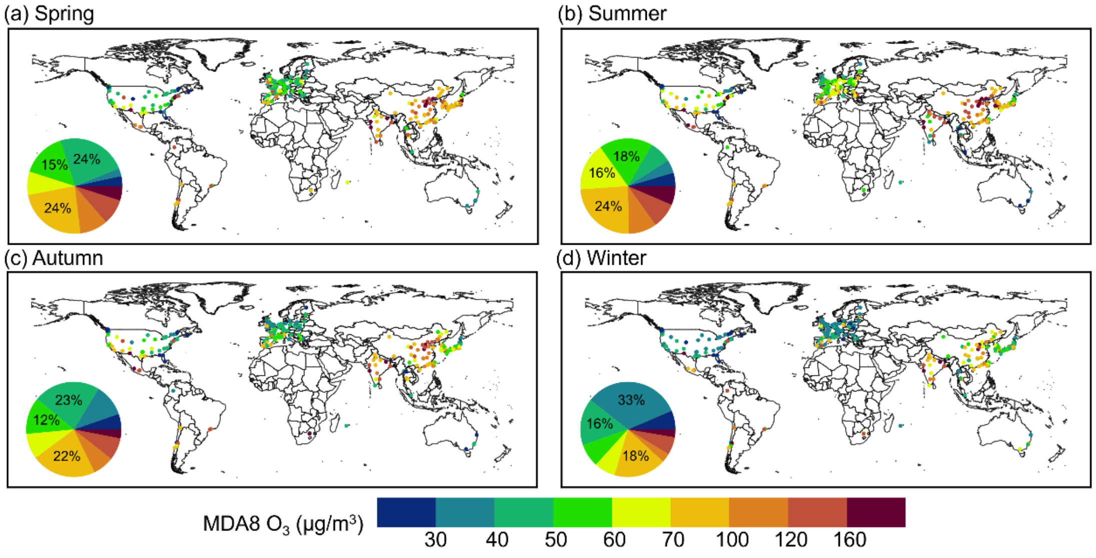

Figure S5. Spatial distribution of quarterly maximum MDA8 O_3 concentrations

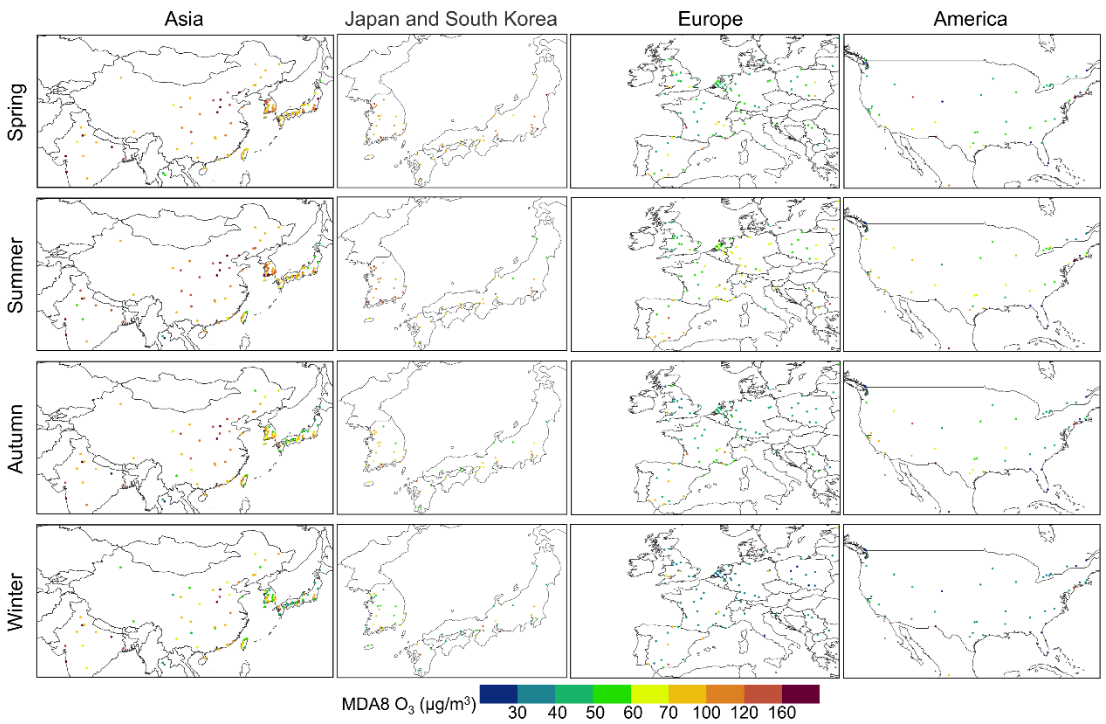

Figure S6. Spatial distribution of quarterly maximum MDA8 O_3 concentrations in Asia, India, Europe, and the United States.

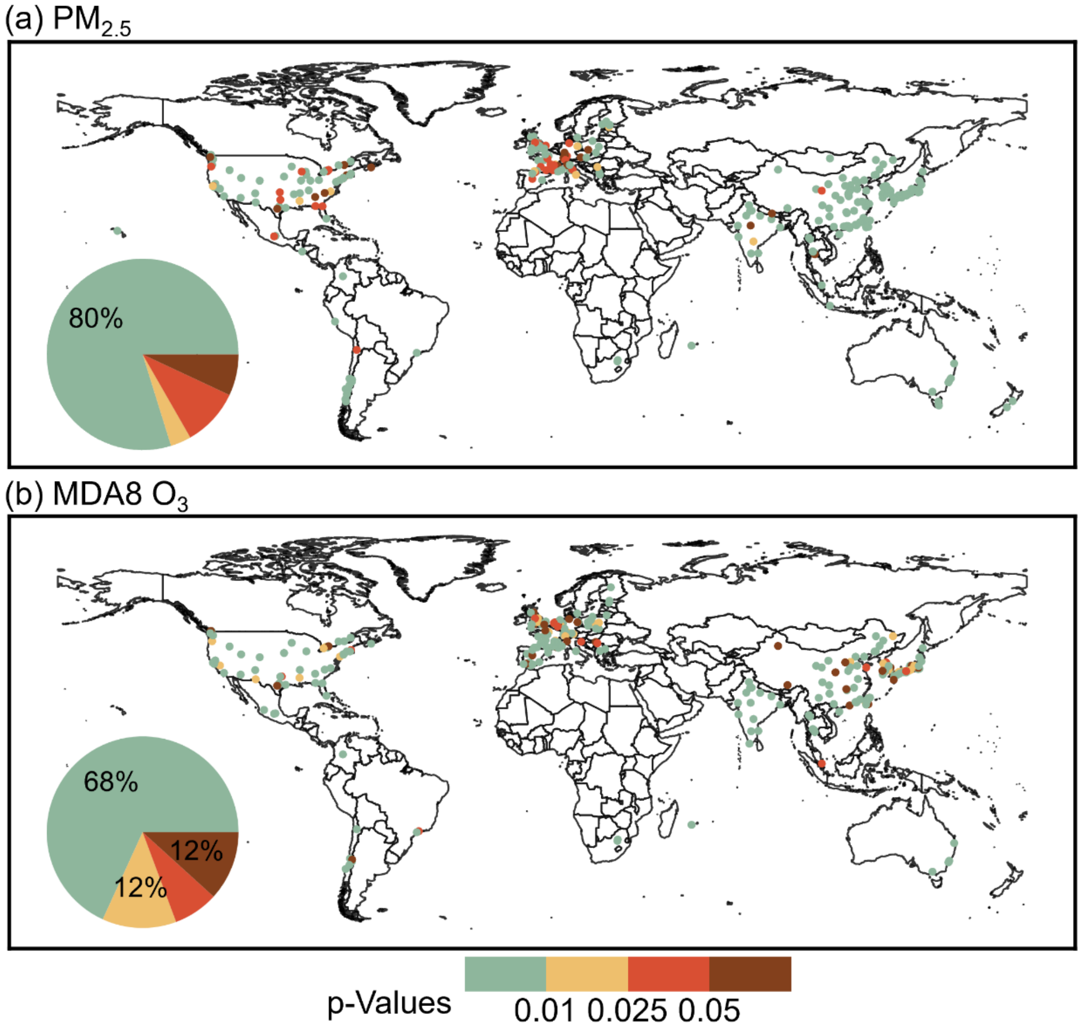

Figure S7. Statistical test for analyzing trends in $PM_{2.5}$ and MDA8 O_3 concentration using the Mann-Kendall method.
